# Customized one-step preparation of sgRNA transcription templates via overlapping PCR Using short primers and its application in vitro and in vivo gene editing

**DOI:** 10.1186/s13578-019-0350-7

**Published:** 2019-10-24

**Authors:** Zheng Hu, Li Wang, Zhaoying Shi, Jing Jiang, Xiangning Li, Yonglong Chen, Kai Li, Dixian Luo

**Affiliations:** 1 The First People’s Hospital of Chenzhou, Affiliated to University of Southern Medical University, Chenzhou, 423000 People’s Republic of China; 2 National & Local Joint Engineering Laboratory for High-throughput Molecular Diagnosis Technology, Affiliated to The First People’s Hospital of Chenzhou, University of South China, Chenzhou, 423000 People’s Republic of China; 3 Translational Medicine Institute, University of South China, Chenzhou, 423000 People’s Republic of China; 4 Department of Biology, Guangdong Provincial Key Laboratory of Cell Microenvironment and Disease Research, Shenzhen Key Laboratory of Cell Microenvironment, Southern University of Science and Technology, Shenzhen, 518055 Guangdong China; 5 Genetalks Bio-tech (Changsha) Limited Liability Company, Changsha, 410013 Hunan China

**Keywords:** Overlap extension PCR, Multiple overlapping primers, sgRNA, Transcription template, Cas9 nuclease

## Abstract

Overlap extension polymerase chain reaction (PCR) is a powerful technology for DNA assembly. Based on this technology, we synthesized DNA templates, which were transcribed into sgRNA in vitro, and further detected their efficiency of purified sgRNAs with Cas9 nuclease. The sgRNAs synthesized by this approach can effectively cleave the DNA fragments of interest in vitro and in vivo. Compared with the conventional method for generating sgRNA, it does not require construction of recombinant plasmids and design of primers to amplify sgRNA core fragment. Only several short primers with overlapped sequences are needed to assemble a DNA fragment as the template of sgRNA. This modified and simplified method is highly applicable and less time-consuming.

**Dear Editor**

Overlap extension PCR efficiently joins different DNA fragments through designing of overlapping primers, which adds an overlap sequence at the end of products and then connect the adjacent fragments with overlap regions. It was first reported that this technology could successfully generate site-directed mutations in a gene [1], and then was widely used in a variety of applications such as to synthesize SARS genomic fragments [2]. At present, this technology has more matured and played an important role in modifying DNA, including site-specific mutations, synthesis of the long fragment of DNA and gene knock out [3, 4].

Clustered Regularly Interspaced Short Palindromic Repeats (CRISPR)/Cas9 system is first found in bacterial and archaea, as an immune defense system to protect against the invasion of viruses and plasmids [5]. It is composed of Cas9 nuclease, CRISPRRNA (crRNA) and trans-activating crRNA (tracrRNA), or a single guide RNA (sgRNA) fused with crRNA and tracrRNA. Distinct from ZFN (zinc finger nucleases) and TALENS (transcription activator-like effectors), CRISPR/Cas9 system identifies the target site using sgRNA and cleaves target sequence to generate DNA double strand breaks (DSBs) with Cas9 nuclease and then initiates different repair mechanisms of non-homologous end joining (NHEJ) or homology-directed repair (HDR) to produce different types of mutation [6]. However, conventional methods of generating sgRNA either direct synthesis sgRNA or requires plasmid construction for PCR amplification to make transcription templates [7], which is either expensive or cumbersome and requires several steps to produces gRNAs.

In the present study, we described a simplified and customized in vitro synthesis approach utilizing multiple overlapping primers to synthesize DNA fragment for six selected gene loci as the transcription templates of sgRNAs by a single-step sequential primer extension under appropriate conditions, and tested the cleave efficiency to targeted loci by using these sgRNAs combined with Cas9 nuclease in vitro and in vivo. The results demonstrate that the sgRNAs produced by overlap extension PCR method could efficiently cleave targeted-DNA by combined with Cas9 nuclease, providing a highly applicable method to prepare sgRAN for targeted locus rapidly.

## Synthesis of sgRNA templates with four overlapping primers

**Fig. 1:**
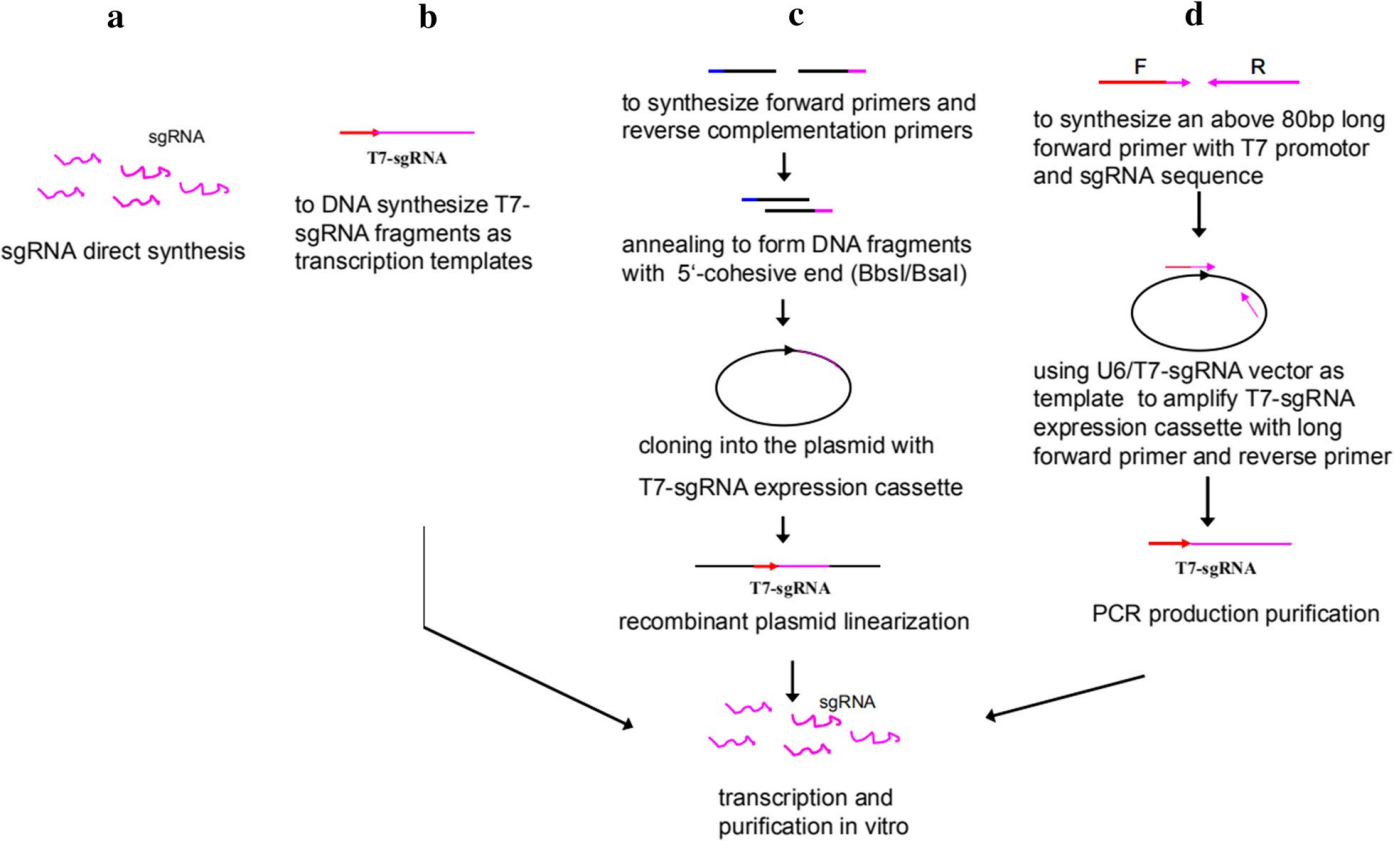
A schematic diagram of the conventional methods to generate sgRNA in vitro. **a** The simple method is direct sgRNA chemical synthesis. The conventional methods to generate sgRNAare in vitro transcription using templates. **b** The direct method to prepare the templates is to DNA synthesize T7-sgRNA fragments as transcription templates. **c** One of methods is to construct a recombinant plasmid with T7-sgRNA backbone sequence to be taken as the sgRNA transcription template. **d** Another method is to take plasmids with T7-sgRNA backbone as PCR templates to amplify transcription templates through using a forward long primer and a reverse primer

Several conventional methods of generating sgRNA have been described and applied in gene functional researches (Fig. 1). Direct sgRNA synthesis at commercial companies is a simple method to customize sgRNAs exception to being expensive (Fig. 1a). The conventional method of generating sgRNA is in vitro transcription using templates. The direct method to make the templates is to synthesize T7-sgRNA DNA fragments at commercial companies as transcription templates (Fig. 1b). To generate sgRNA transcription templates in lab usual either need a plasmid to construct a recombinant plasmid, or need to take plasmids as templates to amplify transcription templates through using a forward long primer and a reverse primer, which either is cumbersome and requires several steps to produces gRNAs or synthesis a long primer above 80 bp each time (Fig. 1c, d).

**Fig. 2:**
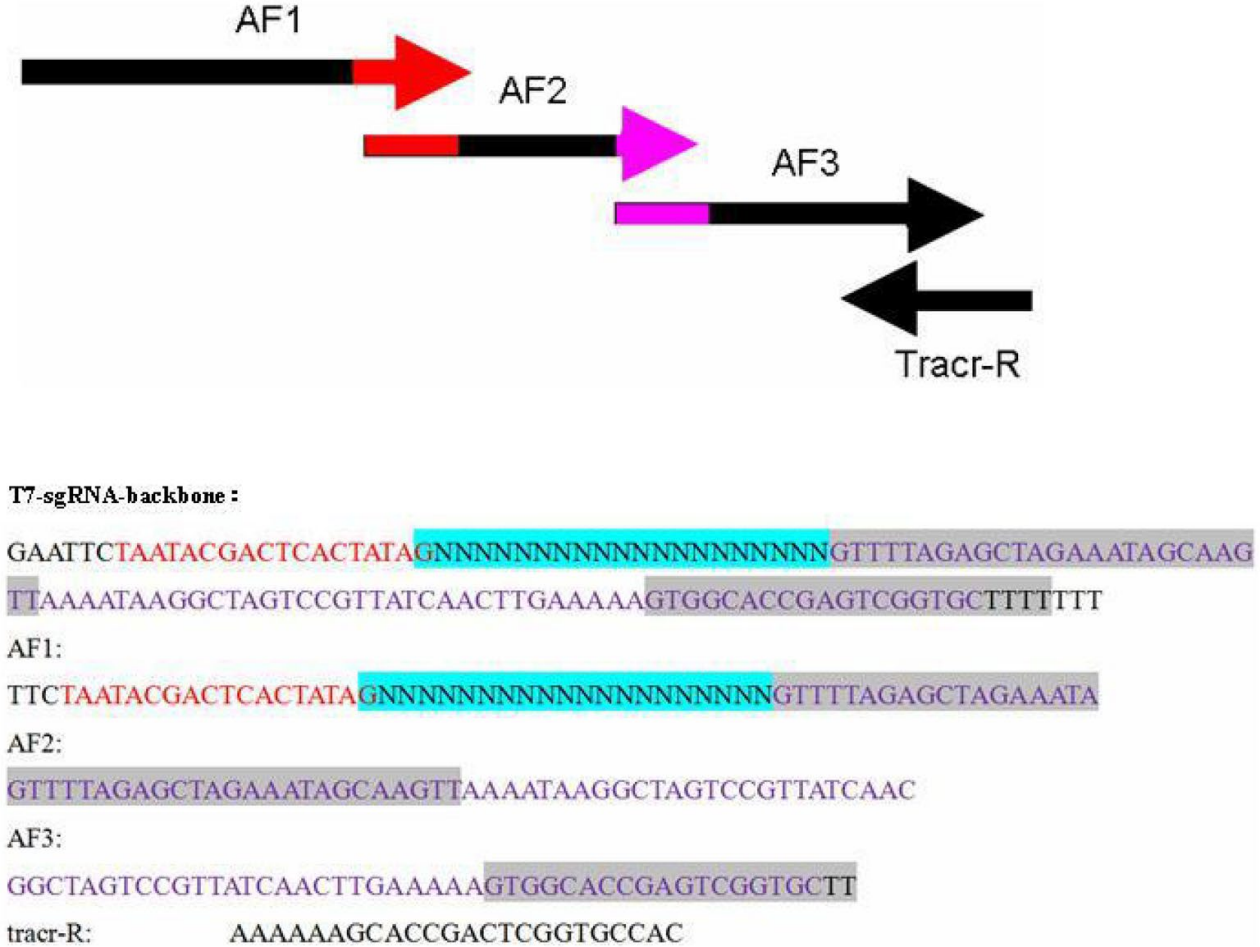
A schematic diagram of overlapping primers amplification. AF1, AF2, AF3 and Tracr-R are overlapping primer. The sgRNA transcription template consists of three parts: T7 promotor (Red marked), the designed sequence of sgRNA (blue background marked), the conserved sequence of sgRNA (the rest of the sequence)

**Fig. 3:**
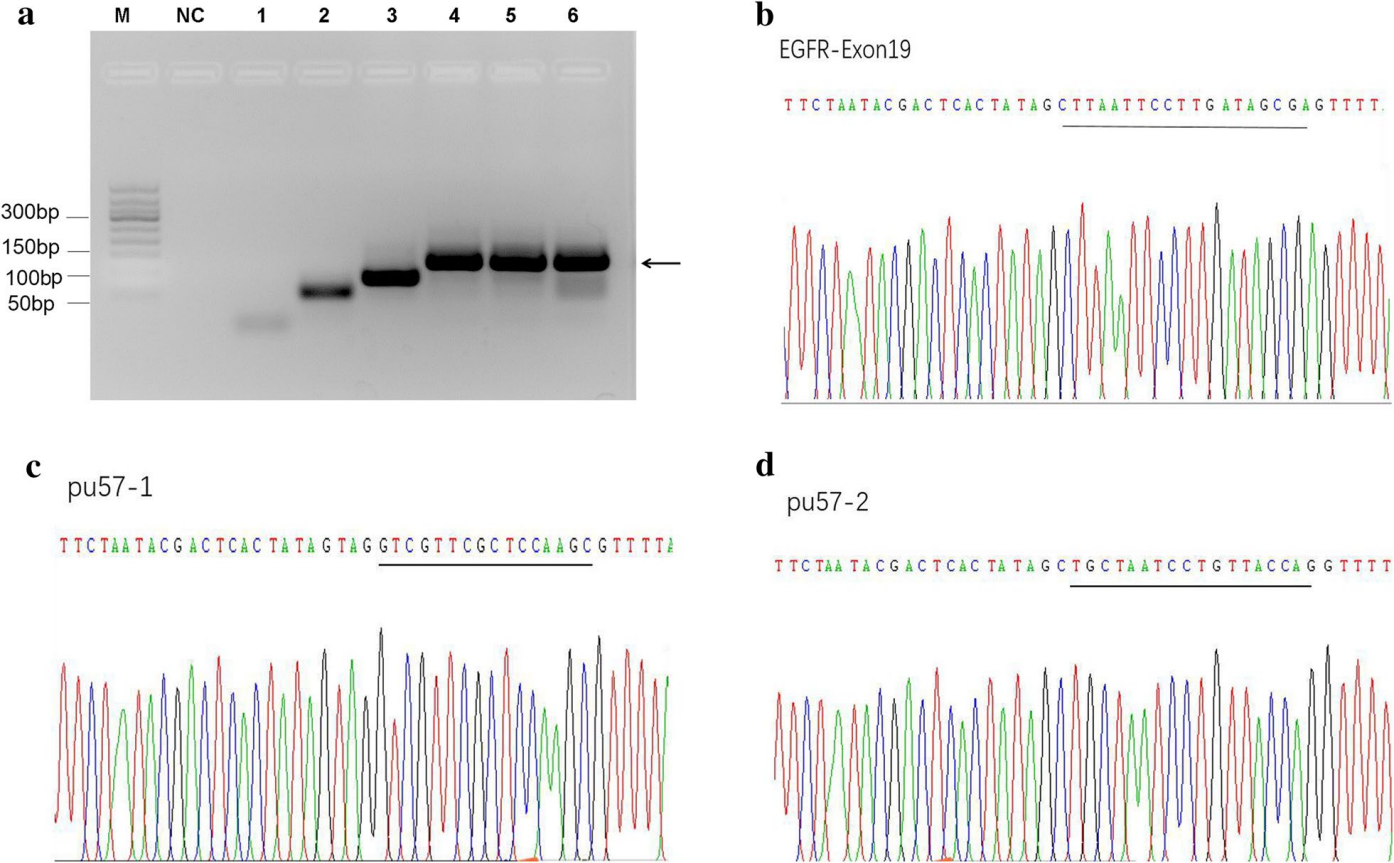
**a** The electropherogram of DNA fragment amplified by the four overlapped primers. M represents 50 bp DNA marker. NC represents the negative control. (1) the tracr-R primer; (2) the two primers : tracr-R and AF3; (3) the three primers: tracr-R, AF3 and AF2; (4, 5, 6) the templates of EGFR, pu57-1 and pu57-2 amplified by four primers respectively. **b**–**d** represent the sequencing results of the transcription template of EGFR-Exon19, pu57-1, pu57-2 respectively. The underlined part represents the designed sequence of sgRNA

The synthesis of sgRNAs transcription templates with overlapping primers consists of four steps and the DNA template includes three parts (Fig. 2). For each gene, we designed four primers that were connected in turn by overlapping parts as shown in Fig. 2. After denaturation, annealing and extension, a final DNA fragment with a fragment size of about 120 bp was synthesized in a PCR instrument, as shown in Fig. 3. The size of the fragment displayed by the electropherogram is consistent with the size of the theoretical primer synthesis, as shown in Additional file 1: Table S3, demonstrating the feasibility of this method.

The sgRNA DNA template synthesized by overlapping primers was shown on electrophoresis photograph (Fig. 3). The size of DNA template fragments of the three genes were around 120 bp. In Additional file 1: Table S3, the final combined fragment labeled by AF1-AF2-AF3-Tracr-R, the longest products, was the template of sgRNA and the size of expected DNA fragments similarly assembled by this means were 122 bp. In order to further verified the sequence of the templates, the PCR products were subcloned into PMD18-T vector and we chose three *E. coli* colonies to confirmed by Sanger’s sequencing. The Fig. 3a–c show the representative sequences of the transcription template of EGFR-Exon19, pu57-1, pu57-2. We demonstrated that the DNA templates of sgRNA synthesized were practicable by using this method. However, it is noteworthy that the design and concentrations of primers in the whole experiment are the key factors affecting the quality of products. In the process of PCR reaction, the concentrations of primers should be adjusted and tested according to the difference in the length of primers. After optimization, the overlapping primers of AF1, AF2, AF3, tracr-R were mixed at the ratios of 50:5:1:50 in the present study, which generated a single DNA fragment nearly with no intermediate products. These optimized parameters ease the synthesis of sgRNA templates with the same quality as commercial kit, but with time saving and less costly.

## Detection of sgRNAs activity and specificity for cleavaged test in vitro

**Fig. 4:**
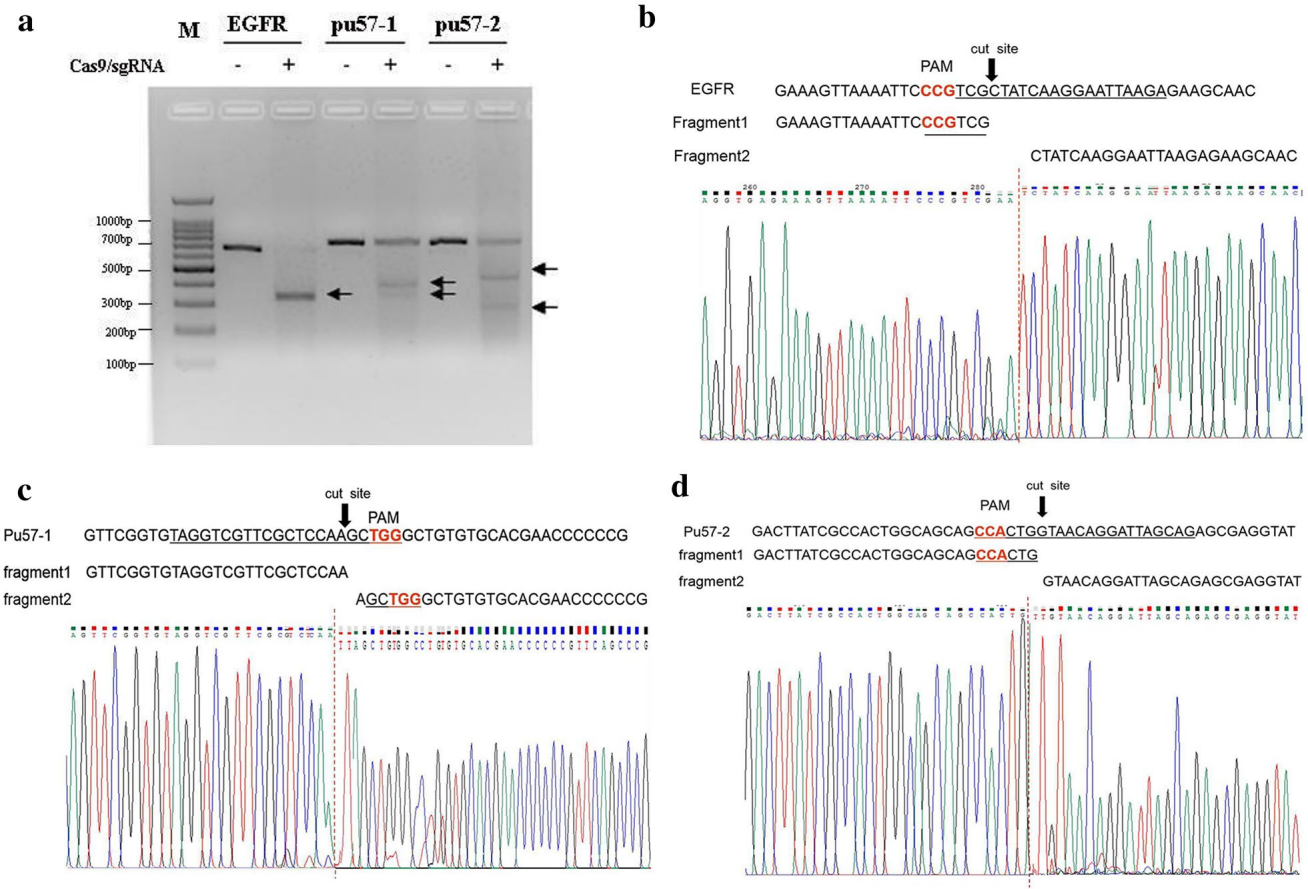
**a** The electropherogram about the result of detection of fragment cleavage site in vitro. EGFR, pu57-1 and pu57-2 represent the PCR fragments with 664 bp, 727 bp and 727 bp length respectively. As the negative control “−” indicates that the gene is not digested by Cas9/sgRNA. “+” indicates that the result of digestion by Cas9/sgRNA. **b**–**d** The representative sequencing results of the cleaved fragments of EGFR, pu57-1 and pu57-2 respectively

We detected the efficiency and specificity of sgRNA combined with Cas9 nuclease in vitro. The agarose gel indicated that EGFR-Exon19 fragment was cut at the target position, leading to the full length of 664 bp cut to two segments with the size about 340 bp and 320 bp. The pu57-1 and pu57-2 were digested into products at the size of 335 bp and 392 bp and the products at the size of 297 bp and 430 bp respectively. These digested fragments were all located at the expected sites after the electrophoresis as shown in Fig. 4a.

Furthermore, the digested products were subsequently confirmed by DNA sequencing analysis. As shown in Fig. 4b–d, the results demonstrated that the three genes were successfully cleaved at the target sites and produced products at the sizes as expected in vitro based on the CRISPR/Cas9 system. The method established in the present study is feasible to explore gene function with site-specific mutation. In general, CRISPR/Cas9 technology has been used extensively to elucidate gene function through the regulation of gene expression [7, 8]. However, the conventional method of obtaining sgRNA is that design primers to amplify DNA fragment containing the target site from plasmid as template and then it is transcribed into sgRNA, which is more cumbersome and need long primers to amplify [9]. Here, the reported method in the present study only requires several short primers, containing approximately 20 bp overlapped sequence for assembling a complete fragment as template of sgRNA. This reported method is faster and more straightforward than conventional one. We demonstrated this approach was an effective tool for functional sgRNA synthesis in vitro. These in vitro synthesized sgRNAs work well for in vitro digestion of target genes, and have applications in mutation analysis, particularly for the cancer hotspot mutations lacking particular restrictive endonucleases.

## Detection of sgRNAs activity and specificity for gene targeting in *X. tropicalis*

**Fig. 5:**
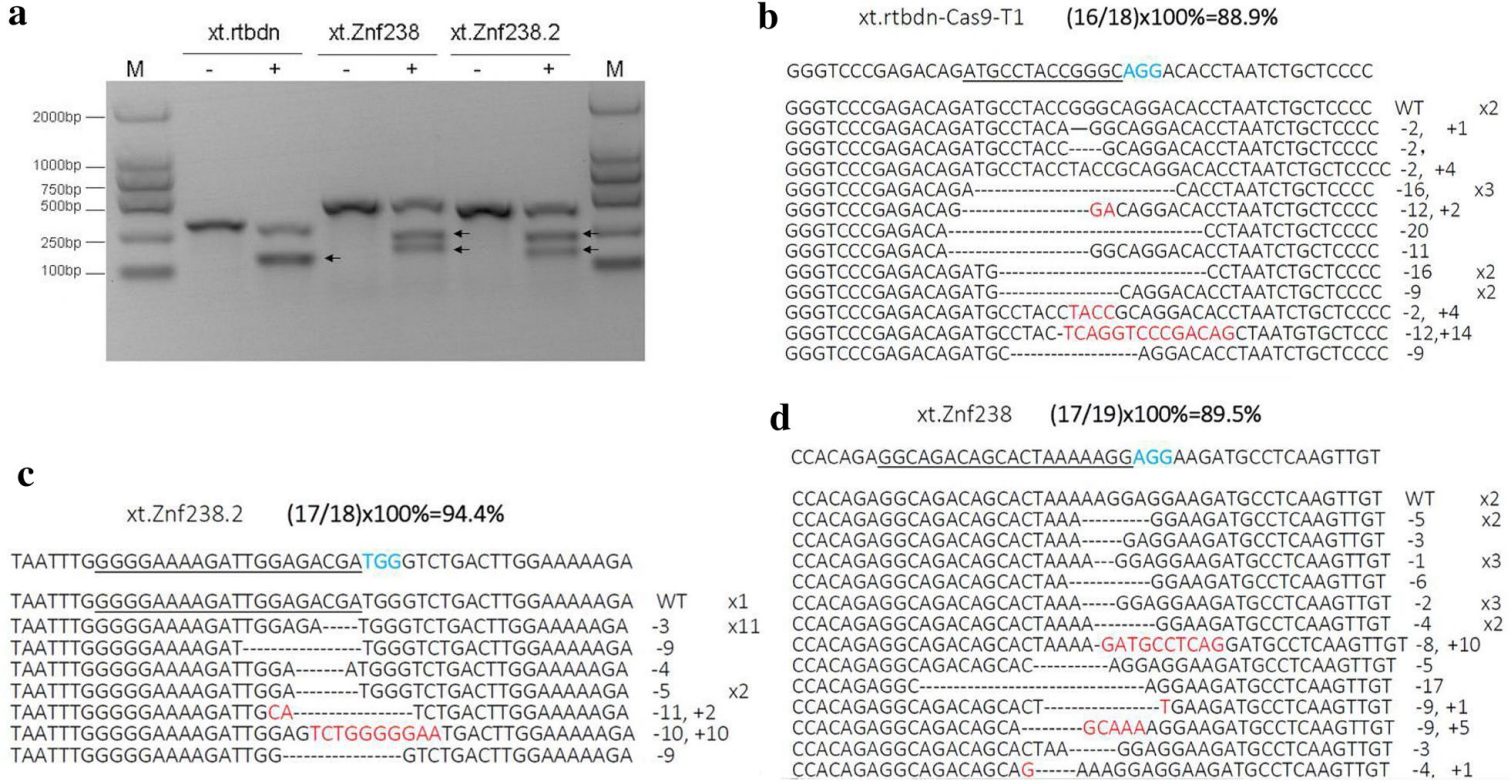
**a** The disruption rate induced by injection of Cas9/sgRNAs into embryos of *X. tropicalis* by T7E1 method assay. “−” represents without T7 enzyme digestion; “+” represents plus T7 enzymedigestion; and “M” represents DNA marker. **b**–**d** The disruption rate induced by injection of Cas9/sgRNAs into embryos of *X. tropicalis* using TA cloning and sequencing analysis. After TA clonging, 20 TA clones were randomly picked up for DNA sequencing, and the disruption rate (%) was calculated based on the results of DNA sequencing

Furthermore, the sgRNAs activity and specificity in vivo were tested using *X. tropicalis*, a frequently used model for study of development as model to assay [7]. Three gene loci, xt.rtbdn, xt.Znf238.2 and xt.Znf238, were selected for these experiments (Additional file 1: Tables S1, S2). Following Cas9 mRNA and sgRNAs co-injected into embryos of *X. tropicalis*, the PCR products of xt.rtbdn, xt.Znf238.2 and xt.Znf238 targeting sequences amplified from embryos genomic DNA were digested with T7 endonuclease I, and the fragments of digestion were analyzed by agarose gel as shown in Fig. 5a. The presence of mutated DNA fragments were clearly observed and identified as there were two segments by T7 enzyme digestion on the agarose gel. To further confirmed the sgRNAs efficiency, another method was applied to assay the targeted disrupted rate after co-injecting Cas9 mRNA/sgRNAs into embryos. The PCR products of xt.rtbdn, xt.Znf238.2 and xt.Znf238 targeting sequences amplified from embryos genomic DNA were used for TA cloning and sequencing. Twenty single colonies were sent to DNA sequencing analysis. The frequency of indels mutations accumulated in the target site of xt.rtbdn, xt.Znf238.2 and xt.Znf238 loci were calculated as described before [7, 8]. As showing in Fig. 5b–d, the frequency of indels mutations in xt.rtbdn, xt.Znf238.2 and xt.Znf238 gene loci are 88.9%, 94.4% and 89.5%, respectively. Together, the results show injection of Cas9 mRNA combined with sgRNAs generated by the four overlapped primers into embryos of *X. tropicalis* efficiently induced targeted mutations in vivo.

Summary, we demonstrated an approach by combining CRISPR/Cas9 gene editing technology with multiple overlapping primers to synthesize DNA fragment as the template of sgRNA to substitute the conventional method. The simplified and optimized method is its simplicity as the sgRNA DNA template can be generated only by the single-step sequential primer extension for rapid production of sgRNA, saving time and money in comparing with purchasing commercial kit. Despite the convenience using commercial kits from companies like as NEB or Thermo Fisher, to generate sgRNAs are expensive and inefficient. Furthermore, we verified that the sgRNA synthesized by this approach was highly efficient and specific in vitro and in vivo. Finally, the present study also demonstrated the application of using synthesized sgRNA in mutation analysis for cancer hotspot mutations.

## Supplementary information

**Additional file 1: Table S1.** The targeting sequences of Cas9/sgRNA. **Table S2.** Overlapping primer sequences for sgRNA transcription templates amplification. **Table S3.** The corresponding products from each step of the sequential primer extension.

## Data Availability

All data generated or analysed during this study are included in this published article [and its additional information files].

## Abbreviations

CRISPR: Clustered Regularly Interspaced Short Palindromic Repeats
ZFN: zinc finger nucleases
TALENS: transcription activator-like effectors
crRNA: CRISPRRNA
tracrRNA: trans-activating crRNA
sgRNA: a single guide RNA
*X. tropicalis*: *Xenopus tropicalis*
DSBs: DNA double strand breaks
NHEJ: non-homologous end joining
HDR: homology-directed repair
