# Supplementary material for: Customized one-step preparation of sgRNA transcription templates via overlapping PCR Using short primers and its application in vitro and in vivo gene editing
Source: Cell Biosci. 2019 Oct 24;9:87. doi: 10.1186/s13578-019-0350-7 (PMC6814055; doi:10.1186/s13578-019-0350-7)
Supplement: Supplementary file 1 — Additional file 1: Table S1. The targeting sequences of Cas9/sgRNA. Table S2. Overlapping primer sequences for sgRNA transcription templates amplification. Table S3. The corresponding products from each step of the sequential primer extension. [file 13578_2019_350_MOESM1_ESM.docx]

Customized One-Step Preparation of sgRNA Transcription Templates via Overlapping PCR Using Short Primers and its Application in vitro and in vivo Gene Editing

Zheng Hu^1,2,3†*^, Li Wang^1,2,3†^, Zhaoying Shi^4^, Jing Jiang^2,3^, Xiangning Li^2,3^, Yonglong Chen^4^, Kai Li^2,3,5*^, Dixian Luo^1,2,3*^

**Additional file**

**Materials and Methods**

Design of target sites and overlapping primers

Three gene loci of EGFR-Exon19, pu57-1, and pu57-2 were selected as sgRNAs target sequence sites for test in vitro, and three X. tropicalis' (Xenopus tropicalis) gene loci of xt.rtbdn-Cas9-T1, xt.Znf238.2 and xt.Znf238 were used as sgRNAs target sequence sites for test in vivo (table.S1). Four overlapping primers including three forward primers and one reverse primer for each gene locus were designed for the assembling of target DNA fragments (table. S2).

Construction of templates of sgRNAs with overlap PCR

The total volume of PCR reaction was 30 *μ*L with 2×Q5 mix ((NEB，#M0494S) 15 *μ*L ), 10 *μ*M AF1 (3.75 *μ*L), 5 *μ*M AF2 (0.75 *μ*L), 1.0 *μ*M AF3 (0.75 *μ*L), 10 *μ*M tracr-R (3.75 *μ*L), DEPC water(6.0 *μ*L). The final concentration of AF1, AF2, AF3 and tracr-R were 1.25 *μ*M, 0.125 *μ*M, 0.025 *μ*M, 1.25 *μ*M, respectively to maximize the generation of template DNAs. The primer extension was carried out using the following conditions: 30 cycles of 95°C for 30 s, 56 °C for 1 min and 72°C for 1 min, followed by a hold at 4 °C. The PCR products were analyzed with 3% agarose gel electrophoresis using 1×TAE buffer at 100 V for 30 min and were photographed by the gel documentation system.

Transcription of sgRNA templates in vitro

The desired DNA products were purified via gel extraction (QIA quick Gel Extraction Kit, Qiagen, #28704), and then approximately 100 ng DNA were used as the template. DNA templates were transcribed into sgRNAs with the T7 in vitro transcription kit (Thermo Scientific，K0441) according the manufacturer's instructions. The products were further purified by RNA purification kit (Qiagen, 217084) under RNase-free environment, which could improve the quality of sgRNA to identify the target site.

Construction of pCS2-Cas9 vector and transcription of Cas9 mRNA in vitro

The codon-optimized Streptococcus pyogenes Cas9 cDNA together with the two attached nuclear localization signals (3×FLAG-NLS-SpCas9-NLS) was synthesized by GenScript and cloned into the pCS2+ vector [1]. The construct was linearized with Not I digestion and transcribed with the mMessage mMachine SP6 Kit (Ambion) to produce capped Cas9 RNA. Transcribed products were further purified with the RNeasy Mini Kit (Qiagen) according to the RNA clean protocol.

Preparation of target genes identified by sgRNA for cleavege test in vitro

We designed two primers pairs of 1 and 2, and of 3 and 4 to amplify the three genes from pUC57 plasmid as the templates to be cleaved by CRISPR/Cas9 system. The primer 1(5'-CGAATGCATCTAGATATCGG-3') and primer 2 (5'-TTACGCCAAGCTTGCATGCA-3') were used to amplify the target gene of EGFR-Exon19 while the DNA fragment amplified by the primers 3 (5'-CACAGAATCAGGGGATAACG-3') and primers 4 (5'-TTCGTTCCACTGAGCGTCAG-3') included the genes of pUC57-1 and pUC57-2. PCR was carried out in a total volume of 30 μL reaction mixtures, followed by 98°C for 2 min, 35 cycles of 98 °C for 10 s, 55 °C for 15 s, 72 °C for 30 s, followed by 72 °C for 2 min. The products were purified and preserved for the subsequent reaction.

Detection of the efficiency and specificity of sgRNA for cleavege test in vitro

We detected the efficiency and specificity of sgRNA combined with Cas9 nuclease in vitro. The reactions contained approximately 500 ng sgRNA, 100 ng Cas9 nuclease, 60 ng target gene, 10 × Cas9 nuclear buffers 2 μL and added RNase-Free water to a final volume of 20 μL. The mixed reaction solution without target gene was incubated at 37 °C for 15 min. Then 60 ng target gene was added into mixture for a further incubation at 37 °C for 2 h. Reaction without sgRNA was also performed as control. The PCR products were divided into two parts. One part of the products was visualized by 2.5% agarose gel at 100V for 30 min, and the other part was used for DNA sequencing analysis to detect the cutting sites of DNA after Cas9/sgRNA digestion.

Co-injected Cas9 mRNA/sgRNAs into one-cell stage embryos of X. tropicalis

X. tropicalis frogs were purchased from Nasco. Ovulation and in vitro fertilization were carried out according to the protocol described previously [2]. The desired amount of Cas9 mRNA and sgRNA in 2 nl was co-injected into one-cell stage embryos as described by Guo et al [1].

Evaluation of gene targeting efficiency in gRNA/Cas9-injected embryos

Forty-eight hours after microinjection (about stage 40), we randomly pooled five healthy embryos from each injection, extracted genomic DNA, amplified the targeted region by PCR. The primers were used for PCR: xt.rtbdn-F: TATGTTAAAGGGGCAGTAGTCTGTT, xt.rtbdn-R: CGGATGTTCTACAGTTACACAAAGC; xt.Znf238.2-F: ggaccagctagacaaaagggatatt, xt.Znf238.2-R: agaggataaaccagaaagtccactg; xt.Znf238-F: ccacctcttttacaaggatcagtta, xt.Znf238-R: agaatctgagggcaatcttatccac. PCR was carried out using 2×Phanta Max Master Mix(Vazyme Biotech,Nanjing,China) with the following cycling conditions for amplification: a cycle of 95 °C for 3 min, 32cycles of 95 °C for 20 s, 58 °C for 15 s and 72 °C for 30 s, and a final cycle of 72 °C for 5 min. The size of PCR fragments for xt.rtbdn, xt.Znf238.2 and xt.Znf238 are 292bp,385bp and 427bp respectively.

Two methods were used for evaluation of gene targeting efficiency. The first method is T7E1 assay according to the protocol described previously [3]. Briefly, PCR product was denatured, reannealed, and digested with T7 endonuclease I (New England BioLabs Inc, Ipswich, MA, USA), as T7 specifically cleaves mis-matched heteroduplex DNA. After digestion, the PCR product was analyzed by agarose gel electrophoresis. The second method is using TA cloning followed by sequencing as described by Guo et al [1]. Briefly, the purified PCR products were cloned into the pMD18-T vector (H101A, Takara, Japan) by TA cloning. Twenty single colonies were randomly picked for DNA sequencing analysis to detect any insertion or deletion (indel) mutations resulting from error-prone non-homologous end joining (NHEJ)-based repair of Cas9-created double-strand breaks. The targeting efficiency was determined by the ratio of mutant to total colonies.

**References**

[1] Guo X, Zhang T, Hu Z, Zhang Y, Shi Z, Wang Q, et al. Efficient RNA/Cas9-mediated genome editing in Xenopus tropicalis. Development. 2014;141:707-14.

[2] Young JJ, Cherone JM, Doyon Y, Ankoudinova I, Faraji FM, Lee AH, et al. Efficient targeted gene disruption in the soma and germ line of the frog Xenopus tropicalis using engineered zinc-finger nucleases. Proc Natl Acad Sci USA. 2011;108:7052-57.

[3] Hu Z, Shi Z, Guo X, Jiang B, Wang G, Luo D, et al. Ligase IV inhibitor SCR7 enhances gene editing directed by CRISPR-Cas9 and ssODN in human cancer cells. Cell Biosci. 2018;8:12.

**Table S1. The targeting sequences of Cas9/sgRNA**

| **Gene locus name** | **Sequence 5' → 3'** |
| --- | --- |
| EGFR-Exon19 | CCGTCGCTATCAAGGAATTAAG |
| pu57-1 | TAGGTCGTTCGCTCCAAGCTGG |
| pu57-2 | CCACTGGTAACAGGATTAGCAG |
| xt.rtbdn | GAGACAGATGCCTACCGGGCAGG |
| xt.Znf238.2 | GGGGAAAAGATTGGAGACGATGG |
| xt.Znf238 | GGCAGACAGCACTAAAAAGGAGG |

Note: The sgRNAs targeting sequences and the PAM sequences of sgRNAs.

**Table S2. Overlapping primer sequences for sgRNA transcription templates amplification**

| **Primer name** | **Sequences 5' → 3'** |
| --- | --- |
| EGFR-AF1 | TTCTAATACGACTCACTATAGCTTAATTCCTTGATAGCGAGTTTTAGAGCTAGAAATA |
| pu57-1-AF1 | TTCTAATACGACTCACTATAGTAGGTCGTTCGCTCCAAGCGTTTTAGAGCTAGAAATA |
| pu57-2-AF1 | TTCTAATACGACTCACTATAGCTGCTAATCCTGTTACCAGGTTTTAGAGCTAGAAATA |
| xt.rtbdn-AF1 | TTCTAATACGACTCACTATAGAGACAGATGCCTACCGGGCGTTTTAGAGCTAGAAATA |
| xt.Znf238.2-AF1 | TTCTAATACGACTCACTATAGGGGAAAAGATTGGAGACGAGTTTTAGAGCTAGAAATA |
| xt.Znf238-AF1 | TTCTAATACGACTCACTATAGGCAGACAGCACTAAAAAGGGTTTTAGAGCTAGAAATA |
| AF2 | GTTTTAGAGCTAGAAATAGCAAGTTAAAATAAGGCTAGTCCGTTATCAAC |
| AF3 | GGCTAGTCCGTTATCAACTTGAAAAAGTGGCACCGAGTCGGTGCTT |
| Tracr-R | AAAAAAGCACCGACTCGGTGCCAC |

Note: All the primers are used in overlap extension PCR. AF1, AF2 and AF3 are forward primers. Tracr-R is reverse primer, and AF2, AF3 and Tracr-R are universal primers that are all involved in the sgRNAs synthesis of genes.

**Table S3. The corresponding products from each step of the sequential primer extension**

| **Double strand converted** | **Expected product** | **Product size** |
| --- | --- | --- |
| AF3-Tracr-R | 5'…aaaagtggcaccgagtcggtgctttttt…3' | 50bp |
| AF2 -AF3-Tracr-R | 5'….aataaggctagtccgttatcaacttga…3' | 82bp |
| AF1-AF2 -AF3-Tracr-R | 5'…gcgagttttagagctagaaatagcaa…3' | 122bp |

Note: AF3-Tracr-R was extended by the underlined overlapped sequences of AF3 and Tracr-R. The next step was extended over one primer bridged by the underlined overlapped sequences. After three rounds of reactions, they became a complete fragment of DNA.
